# Clinical Features and Imaging Findings of Low-Grade Endometrial Stromal Sarcoma: A Retrospective Case Series-Based Analysis

**DOI:** 10.7759/cureus.80507

**Published:** 2025-03-13

**Authors:** Yumiko Miyazaki, Kenji Takata, Tasuku Wakabayashi, Toshimichi Onuma, Hideaki Tsuyoshi, Akiko Shinagawa, Makoto Orisaka, Yoshio Yoshida

**Affiliations:** 1 Obstetrics and Gynecology, University of Fukui, Fukui, JPN; 2 Radiology, University of Fukui, Fukui, JPN

**Keywords:** apparent diffusion coefficient (adc), diffusion-weighted imaging (dwi), endometrial stromal sarcoma, leiomyoma uteri, magnetic resonance imaging

## Abstract

Objective: This case series presents six cases of low-grade endometrial stromal sarcoma (LGESS) and discusses their magnetic resonance imaging (MRI) findings in light of recent advancements in LGESS diagnosis.

Methods: Data from six cases of LGESS treated at Fukui University Hospital between June 2007 and October 2024 were retrospectively analyzed. The clinical behavior, histopathological features, therapeutic approaches, and survival outcomes were evaluated. MRI images were assessed by two radiologists over multiple parameters that were considered specific to the LGESS.

Results: Among the six patients with LGESS, four patients had tumors located within the myometrium, whereas two patients had tumors located in the submucosal layer. All cases were classified as International Federation of Gynecology and Obstetrics Stage Ⅰ, and no recurrence was observed during follow-up. On MRI, all six tumors exhibited high signal intensity on T2-weighted images (T2WI), compared to the myometrium, and high signal intensity on diffusion-weighted images (DWI), compared to the endometrium. In four of the five evaluable cases, the mean apparent diffusion coefficient (ADC) value was 0.86 × 10⁻³ mm²/s (range: 0.81-0.99).

Conclusion: Both clinically and based on imaging findings, distinguishing between LGESS and rare leiomyoma variants is challenging. MRI findings, including high signal intensity on T2WI and DWI as well as low ADC values, may prove valuable in differentiating these two entities.

## Introduction

Low-grade endometrial stromal sarcoma (LGESS), a rare uterine stromal tumor, accounts for 0.2% of all uterine malignancies and is the second most common uterine sarcoma [1]. The annual incidence of endometrial stromal sarcoma (ESS) is approximately 0.30 per 100,000 women, and ESS primarily affects those in the perimenopausal or postmenopausal stages [2]. In cases of LGESS extending to the endometrium, deep curettage of the endometrium may be efficacious for preoperative diagnosis [3]. Conversely, when the tumor is located in the myometrium, obtaining a histopathological diagnosis prior to surgery can be challenging [3].

Magnetic resonance imaging (MRI) enables differentiation between uterine leiomyomas and uterine sarcomas. MRI findings suggestive of sarcoma, including ill-defined tumor margins, intratumoral hemorrhagic necrosis, early contrast enhancement, and diffusion restriction on diffusion-weighted imaging (DWI; low apparent diffusion coefficient (ADC)), have been reported [4]. Based on a meta-analysis of 18 studies, the Food and Drug Administration reported that the risk of uterine sarcoma in patients diagnosed with leiomyoma was 0.28% [5]. In particular, LGESS is often diagnosed initially as uterine leiomyoma on preoperative imaging and later confirmed postoperatively. In recent years, laparoscopic hysterectomy has been widely performed for uterine fibroids, and the number of cases of LGESS diagnosed after such procedures has increased. Notably, studies have reported that the risk of recurrence increases when the uterus is morcellated intraperitoneally during laparoscopic surgery [6,7]. Therefore, preoperatively distinguishing between LGESS and uterine fibroids is of critical importance for selecting the most appropriate surgical approach. However, advancements in imaging diagnostics for LGESS remain limited.

In this paper, we present six cases of LGESS encountered in our department to contribute to a more comprehensive understanding of the preoperative MRI findings associated with LGESS.

## Materials and methods

Clinical data

We analyzed seven cases of LGESS at Fukui University Hospital between 2007 and 2024. Two experienced pathologists reviewed and confirmed surgical specimens. One case was excluded due to the presence of concomitant epithelial endometrial carcinoma of a different histological type. This retrospective study was approved by our institutional review board at the University of Fukui Hospital (approval number: #20150016), which waived the requirement for informed consent. Clinical and follow-up data were obtained from medical records. The collected information included parity, symptoms, tumor locations, imaging findings (restricted to data collected from 2004 to 2024), treatment modalities, and survival status. Following surgery, patients underwent follow-up examinations, including pelvic and transvaginal ultrasound examinations, every three months for the first two years, every six months from the third to the fifth year, and annually thereafter. Additionally, computed tomography scans were performed once a year. The final censoring date for assessing survival time was December 31, 2024.

MRI protocol and image analysis

All MRI examinations were conducted in the supine position using a pelvic phased array coil. MRI data were acquired using 1.5 T Signa Excite (GE Healthcare, Milwaukee, WI, USA) from 2007 to 2010. From 2020 to 2022, 1.5 T Ingenia CX (Philips, Amsterdam, The Netherlands) and 1.5 T Optima MR 360 (GE Healthcare, Milwaukee, WI, USA) were used, while 3 T Ingenia Elition X (Philips, Amsterdam, The Netherlands) was utilized in 2024. DWI images were recorded for five of the six patients. The high b-values ranged between 800 and 1,000 s/mm^2^. Two board-certified radiologists with nine and 14 years of experience, respectively, independently re-evaluated the pretreatment MRI features of LGESS with reference to previously published studies [8,9]. The following qualitative features were assessed: on T2-weighted images (T2WI) [1], worm-like nodular extensions [2,4], low signal intensity (SI) zones within the tumor [3], low SI margins surrounding the intramuscular tumor (low SI rim) [5,10], cystic and/or necrotic changes [8], and SI higher than that of the outer myometrium [10]. When contrast-enhanced MRI was available, heterogeneous tumor enhancement was observed [4]. When DWI was available, the following features were assessed: SI of the solid components in relation to the signal of the uterine endometrium [7] and the ADC value in the solid component [8]. A circular region of interest (ROI) measuring 100 mm² was positioned in the targeted areas with the lowest ADC values. Precautions were taken to avoid hemorrhage, necrosis, and major vascular structures. A minimum of three measurements were obtained from adjacent slices and averaged.

Statistical analysis

Statistical analyses were performed using GraphPad Prism 10 (GraphPad Software, San Diego, CA, USA). Group differences were assessed using the t-test, with p < 0.05 considered statistically significant. Reader agreement on imaging features and interobserver variability were evaluated using Cohen’s kappa statistic. Agreement levels were interpreted as follows: <0.20, slight; 0.21-0.40, fair; 0.41-0.60, moderate; 0.61-0.80, substantial; and 0.81-1.00, near-perfect [11].

## Results

Clinical features and treatment outcomes

**Figure 1:**
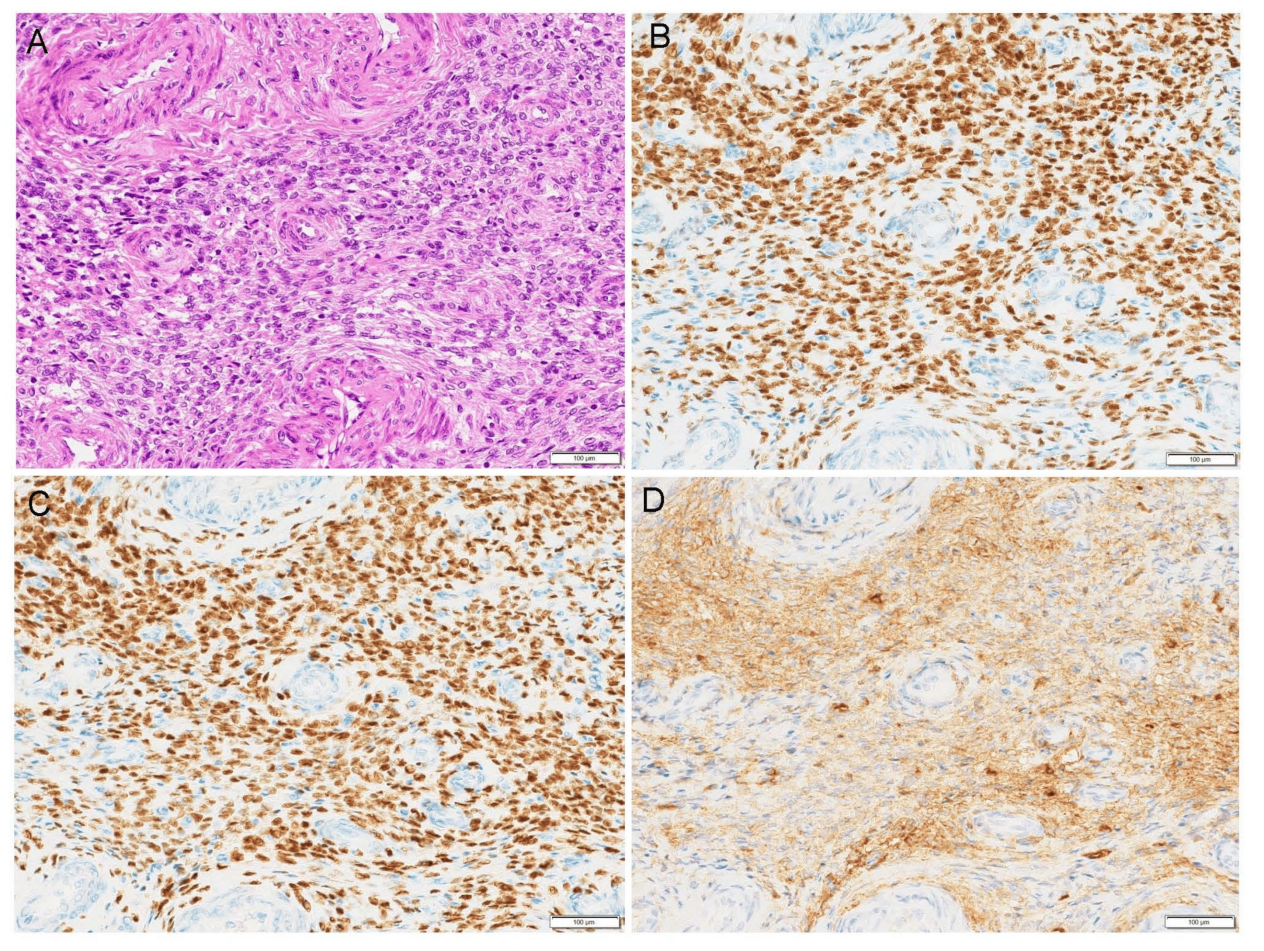
Microscopic features of low-grade endometrial stromal sarcoma (A) Neoplastic cells exhibiting uniform oval-to-spindle-shaped nuclei and morphologically resembling the stroma of the endometrium in the proliferative phase. (B–D) Immunohistochemical analysis demonstrates positive staining for estrogen receptor (B), progesterone receptor (C), and CD10 (D). Scale bar: 100 μm

Age at first consultation ranged from 37 to 58 years (median age 46 years). Two patients presented with hypermenorrhea, one had abnormal uterine bleeding, and three were asymptomatic. The masses were located intramural in four cases and submucosal in two cases. The mean tumor diameter was 52 mm (range: 30-103 mm). One patient (case 5) was diagnosed with LGESS via a biopsy prior to treatment, while the remaining patients were preoperatively diagnosed with uterine leiomyoma (Table 1) and later confirmed to have LGESS based on surgical specimens (Figure 1A). Furthermore, among the six patients, one patient underwent transcervical resection (case 3), one underwent transabdominal tumor enucleation (case 4), and one underwent laparoscopic total hysterectomy and bilateral salpingectomy (case 6) as the initial surgical intervention. All three patients required a second surgery to achieve complete remission. Immunohistochemistry was performed in five cases, all of which demonstrated positivity for the estrogen receptor (ER) (Figure 1B), progesterone receptor (PgR) (Figure 1C), and CD10 (Figure 1D). The median follow-up period was 40 months (range: 1-205 months), during which no recurrence or metastasis was observed. The details are summarized in Table 1.

**Table 1:** Clinical and pathological features in six patients with LGESS AUB: abnormal uterine bleeding; BS: bilateral salpingectomy; BSO: bilateral salpingo-oophorectomy; ER: estrogen receptor; FIGO: International Federation of Gynecology and Obstetrics; IHC: immunohistochemistry; LVSI: lymphovascular space invasion; LGESS: low-grade endometrial stromal sarcoma; N/A: not available; NED: no evidence of disease; PgR: progesterone receptor; TAH: total abdominal hysterectomy; TLH: total laparoscopic hysterectomy; TCR: transcervical resection

Case	Age	Parity	Symptoms	Size (mm)	LVSI	IHC			FIGO	Pretreatment diagnosis	Surgical procedures	Recurrence	Follow-up (months)
						ER	PgR	CD10					
1	47	G2P2	No symptoms	60	+	N/A	N/A	N/A	ⅠB	Leiomyoma (myxoid degeneration)	TAH+BSO	NED	205
2	58	G0	No symptoms	30	-	+	+	+	ⅠA	Cellular leiomyoma	TAH+BSO	Lost to follow-up	110
3	35	G1P1	Hypermenorrhea	33	-	+	+	+	ⅠA	Submucosal leiomyoma	TCR, TAH+BSO	Lost to follow-up	32
4	37	G1P1	No symptoms	103	-	+	+	+	ⅠB	Leiomyoma	Tumor resection, TAH+BSO	NED	48
5	45	G3P3	Abnormal genital bleeding	31	-	+	+	+	ⅠA	LGESS	TAH+BSO	NED	31
6	52	G0	Hypermenorrhea	58	+	+	+	+	ⅠB	Cellular leiomyoma	TLH+BS, oophorectomy	NED	1

Characteristics of radiological images

**Figure 2:**
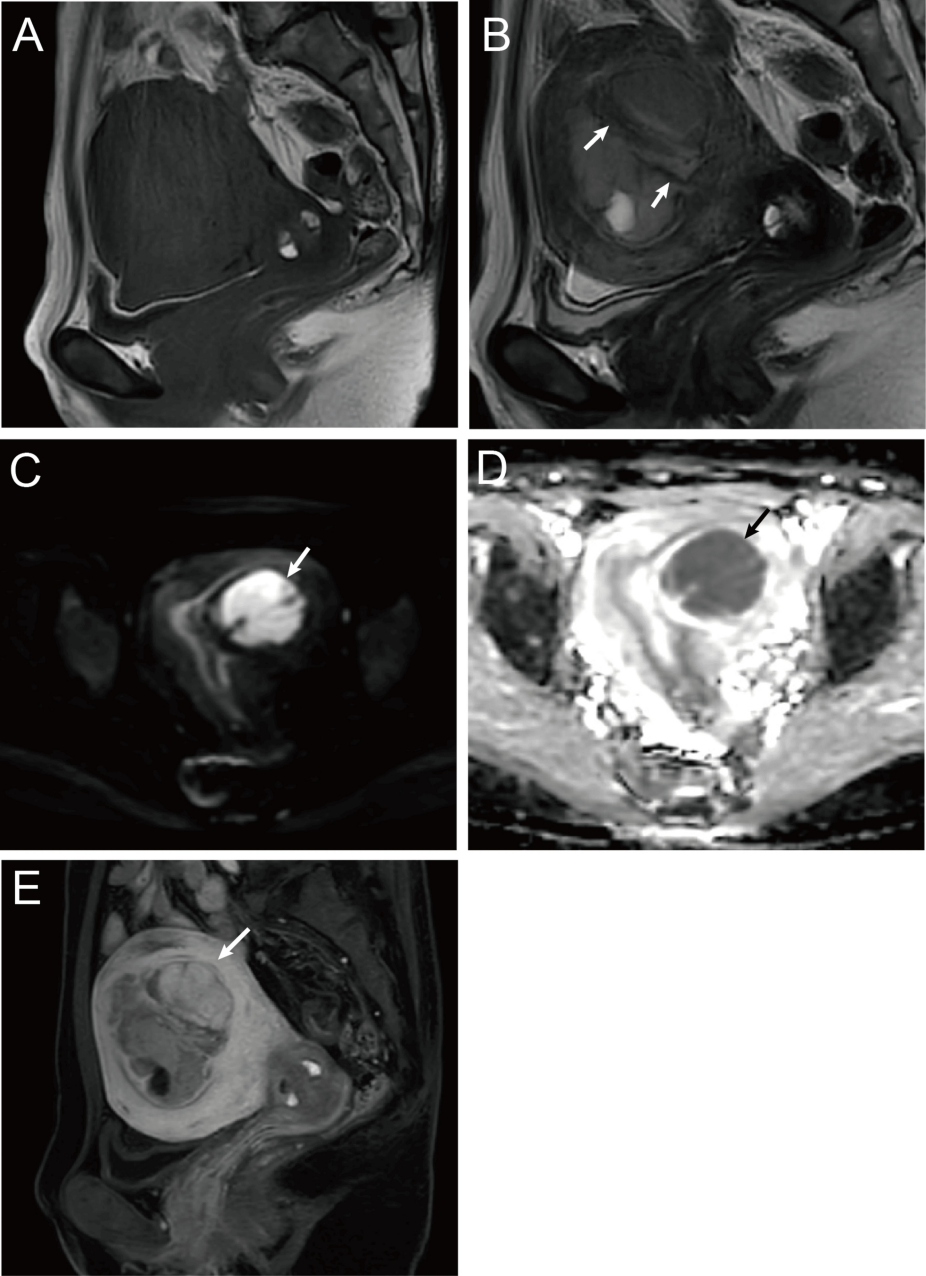
Fifty-two-year-old woman with LGESS (case 6) A mass was found in the uterine wall showing isointensity on T1WI (A) and heterogeneous hyperintensity with hypointense bands (arrows) on T2WI (B). Diffusion-weighted imaging (DWI) (C) and apparent diffusion coefficient (ADC) map (D) showing hyperintense and hypointense masses, respectively (arrows). Sagittal contrast-enhanced T1WI with fat suppression (E) showed a heterogeneous enhanced mass. T1WI: T1-weighted imaging; T2WI: T2-weighted imaging; LGESS: low-grade endometrial stromal sarcoma

**Figure 3:**
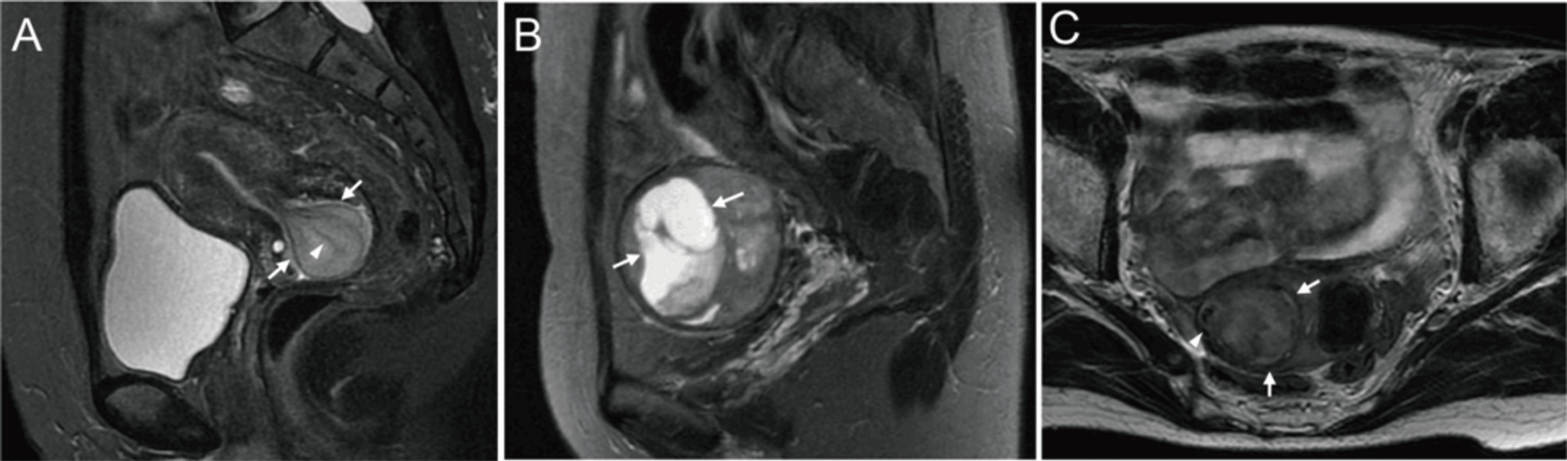
Magnetic resonance imaging findings in LGESS in representative cases A submucosal lesion is evident on sagittal T2WI (A) and protrudes into the vaginal canal (arrows); notably, an intramass band-like structure with low signal intensity (arrows) was observed (case 3). Sagittal T2WI (B) reveals an oval, well-defined, heterogeneously hyperintense myometrial mass with cystic areas (arrows; case 1). On axial T2WI (C), a T2 high-signal mass surrounded by a low-signal rim (arrows) exhibits nodular invasive growth (arrows; case 2). T2WI: T2-weighted imaging; LGESS: low-grade endometrial stromal sarcoma

**Figure 4:**
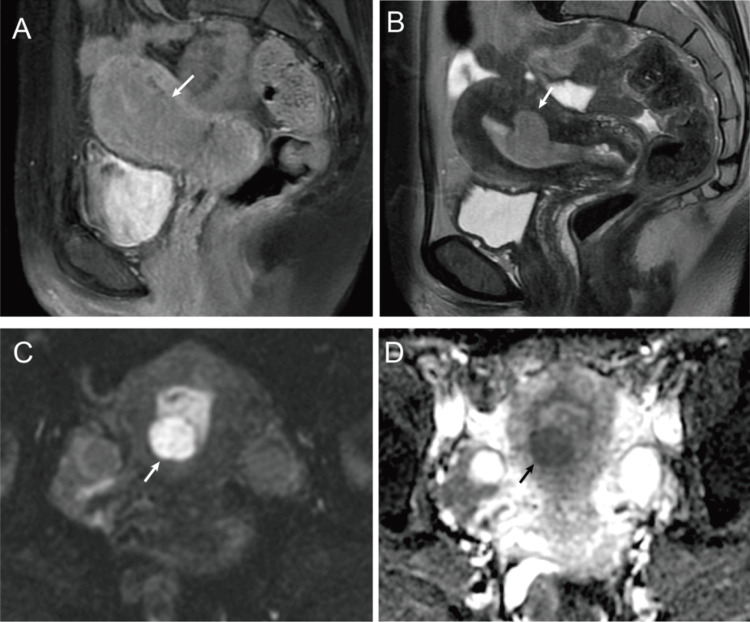
Forty-five-year-old woman with LGESS (case 5) A mass was located in the uterine posterior wall (arrow), showing isointensity on T1WI (A) and hyperintensity on T2WI (B). This mass showed high signal intensity upon diffusion-weighted MRI (C) with a low ADC value (arrows) (D). LGESS: low-grade endometrial stromal sarcoma; T1WI: T1-weighted imaging; T2WI: T2-weighted imaging; MRI: magnetic resonance imaging; ADC: apparent diffusion coefficient

**Figure 5:**
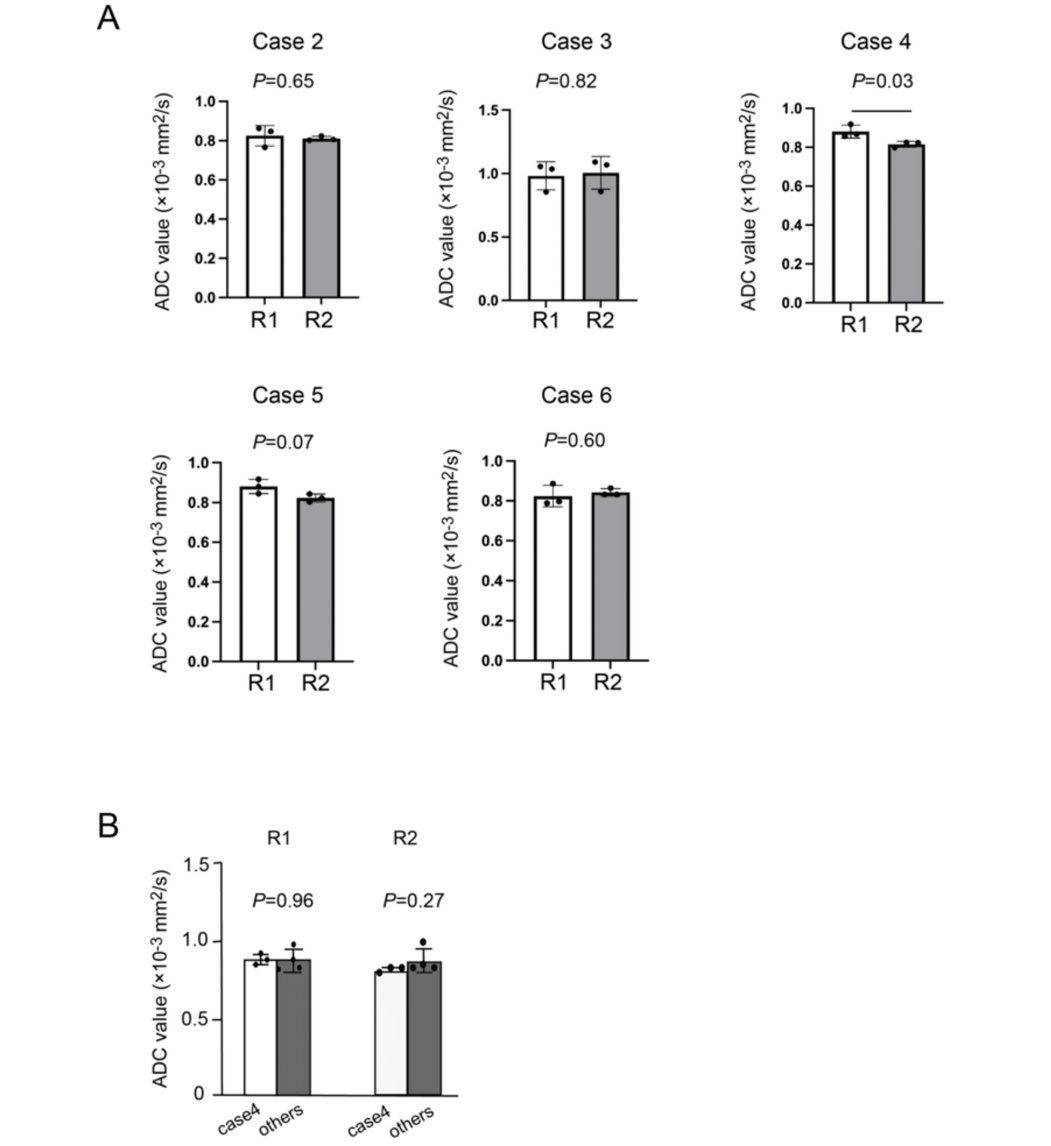
Evaluation of average ADC values by two readers (A) The average ADC values for each case from the two readers were analyzed using a t-test, with p < 0.05 considered indicative of a significant difference. Except for case 4, no significant differences were observed in the average ADC values between the two readers. (B) No significant difference was detected between case 4 and the other cases (cases 2, 3, 5, and 6). R: reader: ADC: apparent diffusion coefficient

The inter-reader concordance rate for findings such as worm-like nodular extension, intramural low SI bands (Figures 2B, 3A), and low SI rims (Figure 3C) ranged between 50% and 66.7%. Conversely, the concordance rate for findings such as cystic and/or necrotic changes (Figure 3B), heterogeneous tumor enhancement, SI higher than that of the outer myometrium (Figures 2B, 3A, 3C, 4B), and high SI on DWI (Figures 2C, 4C) was 100% (Table 2). All six tumors exhibited a higher SI than the outer myometrium, and high SI was observed in all five patients who underwent DWI. The ADC value in the five measurable cases was 0.86 × 10⁻³ mm²/s (range: 0.81-0.99), with four cases demonstrating ADC values below 0.9 × 10⁻³ mm²/s. Except for that in case 4, no significant differences were observed in the average ADC values between the two readers (Figure 5A). No patient exhibited intratumoral hemorrhage or pelvic lymph node swelling.

**Table 2:** Magnetic resonance imaging features in six patients with LGESS LGESS: low-grade endometrial stromal sarcoma; ADC: apparent diffusion coefficient; DWI: diffusion-weighted imaging; FIGO: International Federation of Gynecology and Obstetrics; κ: kappa coefficient; R: reader; SI: signal intensity; N/A: not available

Case	Tumor location	Worm-like nodular extension		Intramural low SI band		Low SI rim		Cystic and/or necrotic change		Heterogeneous tumor enhancement		Higher SI than outer myometrium		DWI high signal		ADC value (×10^-3 ^mm^2^/s) (median, range)	
		R1	R2	R1	R2	R1	R2	R1	R2	R1	R2	R1	R2	R1	R2	R1	R2
1	FIGO type 6	+	−	+	+	+	+	+	+	+	+	+	+	N/A	N/A	N/A	N/A
2	FIGO type 3	−	−	+	+	+	+	−	−	+	+	+	+	+	+	0.82 (0.76-0.86)	0.81 (0.80-0.82)
3	FIGO type 0	−	−	+	+	+	+	−	−	N/A	N/A	+	+	+	+	0.98 (0.85-1.05)	1.00 (0.85-1.09)
4	FIGO type 6	+	−	+	−	+	−	−	−	N/A	N/A	+	+	+	+	0.88 (0.85-0.91)	0.81 (0.79-0.82)
5	Submucosal	−	−	−	+	−	+	−	−	−	−	+	+	+	+	0.88 (0.84-0.91)	0.82 (0.80-0.84)
6	FIGO type 2	+	−	+	+	+	+	+	+	+	+	+	+	+	+	0.82 (0.78-0.88)	0.84 (0.83-0.86)
Inter-reader concordance (%)		50		66.7		66.7		100		100		100		100		N/A	
κ		0		-0.2		-0.2		1		1		N/A		N/A		N/A	

## Discussion

Both LGESS and uterine leiomyomas can manifest during the premenopausal and perimenopausal periods [1], frequently exhibit similar clinical progressions, and express ER and PgR [1]. Therefore, tumor dimensions can be reduced with GnRH analog-based treatment, which makes differentiation more challenging. In this investigation, we examined the clinical course and imaging findings of six cases of LGESS. In two of the six cases, the patients had submucosal tumors and presented with genital bleeding. The remaining four cases were asymptomatic. Only one patient with a submucosal tumor underwent preoperative endometrial biopsy and was diagnosed preoperatively as LGESS on histological examination. These findings underscore the difficulty in diagnosing LGESS based solely on the clinical course and thereby emphasize the critical role of imaging in diagnosis.

Based on MRI, substantial knowledge has been accumulated to facilitate the differentiation of uterine leiomyosarcoma from leiomyoma. However, owing to the rarity of the tumor, there is limited MRI evidence for LGESS. The characteristic MRI findings of LGESS include worm-like or nodular extensions, low SI bands within the tumor on T2WI, and low SI margins on T2WI [8]. In this investigation, two radiologists evaluated the presence or absence of these findings in six cases of LGESS. At least one of these qualitative findings was observed in all six cases (Table 2). However, the interobserver agreement rate was low (50%-66.7%), indicating potential challenges in diagnostic reproducibility. In contrast, certain MRI features, including cystic and/or necrotic change, heterogeneous tumor enhancement, SI higher than that in the outer myometrium, and high DWI signals, demonstrated a concordance rate of 100%, indicating high inter-reader reproducibility. However, these features are not entirely specific to LGESS. For example, cystic changes within a tumor are also observed in leiomyomas with cystic degeneration [4]. Similarly, necrotic changes and heterogeneous tumor enhancement are also found in uterine leiomyosarcomas and degenerated leiomyomas [4,12]. Moreover, SI higher than that of the outer myometrium and high DWI signals can be present in cellular leiomyomas, leiomyosarcomas, and degenerated leiomyomas [12]. Therefore, while these findings exhibit high reproducibility, their specificity for differentiating LGESS from other uterine tumors remains limited.

The presence of high signal intensities on DWI and low ADC values in the solid components of a mass reflects high cellular density and has been observed in some malignant tumors, such as sarcoma or carcinoma [12]. In cases of atypical uterine masses, moderate-to-high signal intensities on T2WI, signal intensities equal to or higher than that of the endometrium on DWI, and ADC values < 0.9 have been proposed as indicators of malignancy [3]. Moreover, the utility of ADC values in differentiating LGESS from rare variants of leiomyomas has been emphasized [8,13]. Although DWI and ADC are valuable modalities for differentiating uterine sarcomas from degenerative leiomyomas, distinguishing between uterine sarcomas and cellular leiomyomas remains challenging [12]. Several studies have attempted to distinguish between uterine sarcomas and cellular leiomyomas. For instance, Himoto et al. reported that imaging features such as low SI bands within the tumor, cystic/necrotic changes, and patchy signal loss on T2WI-reflecting the slow but invasive pathological behavior of LGESS-may aid in distinguishing LGESS from rare leiomyoma variants [8]. Furthermore, Wang et al. demonstrated that assessing tumor morphology (round/oval vs. irregular), border definition (clear vs. unclear), and ADC values may enhance the accuracy of differential diagnosis between leiomyoma and leiomyosarcoma [14]. Expanding on these criteria may help improve diagnostic confidence in differentiating these lesions. Thus, integrating DWI and ADC findings with other diagnostic indicators is crucial when differentiating LGESS from cellular leiomyomas. In this study, Cases 2 and 6 were preoperatively diagnosed with cellular leiomyoma (Table 1). However, it was considered that combining the ADC values with findings from T2WI could facilitate the diagnosis of LGESS (Table 2). In this study, among the six cases for which ADC values could be measured, four exhibited ADC values < 0.9 × 10⁻³ mm²/s (Table 2). Apart from case 4, the average ADC values demonstrated minimal variation between radiologists (Figure 5A), which indicates the high reproducibility of the ADC measurements. In case 4, a significant difference in ADC values was observed between readers. However, the statistical comparison between case 4 and the other cases (cases 2, 3, 5, and 6) indicated no significant difference, suggesting that the ADC values of case 4 fell within the range of other LGESS cases (Figure 5B). Therefore, the significant difference observed between R1 and R2 in case 4 was likely due to inter-reader variability rather than an inherent difference in the lesion itself. Combined, these results indicate that high DWI SI and low ADC values may contribute to an accurate imaging diagnosis of LGESS.

In cases in which LGESS is preoperatively misdiagnosed as a uterine fibroid, the patient may undergo laparoscopic total hysterectomy or laparoscopic tumor resection, during which morcellation can be performed in the abdominal cavity to extract the uterus. This procedure may result in the dissemination of tumor tissue within the abdominal cavity and thereby increase the risk of recurrence. A meta-analysis of four observational studies among patients with uterine sarcoma demonstrated that morcellation was associated with significantly higher recurrence and mortality rates than cases without morcellation [15]. Furthermore, studies have reported a significant decrease in disease-free survival over a five-year period when morcellation was performed for LGESS [6]. Although most international guidelines do not recommend adjuvant hormonal therapy for patients with International Federation of Gynecology and Obstetrics Stage I LGESS, postoperative hormonal therapy is advised in cases involving morcellation [7]. Consequently, morcellation within the abdominal cavity should be avoided when LGESS is suspected. Safe in-bag morcellation of uterine fibroids may mitigate many morcellation-associated complications [5]. Notably, not all risks, such as the potential leakage of bag contents into the abdominal cavity, can be entirely eliminated [16]. To date, there has been no clear evidence that in-bag morcellation reduces the risk of recurrence in LGESS. Given the rarity of LGESS, long-term prognostic data on patients who have undergone in-bag morcellation remain limited. Therefore, sound conclusions regarding its impact on recurrence risk cannot be drawn. As more cases accumulate in the future, the effect of in-bag morcellation on recurrence risk reduction may become clearer.

In cases where LGESS is preoperatively misdiagnosed as a benign tumor, ovarian preservation may be proposed as a treatment option. Although it does not affect the overall survival rates, ovarian preservation is associated with an elevated risk of recurrence. A systematic review of 786 LGESS patients demonstrated that the rate of tumor recurrence was significantly higher (odds ratio 2.7) in patients who underwent ovarian preservation than in those who underwent bilateral salpingo-oophorectomy [17]. The number of reports on hormone receptor expression in recurrent LGESS lesions is limited. However, the high response rate to hormone therapy in recurrent cases [18] suggests that hormone receptors are also expressed in these lesions. This notion supports the hypothesis that the increased recurrence risk associated with ovarian preservation may be driven by hormonal influences on residual tumor cells. Therefore, when LGESS cannot be ruled out preoperatively, the decision to preserve the ovaries should be carefully evaluated, ensuring that patients receive comprehensive information about recurrence risk. Ovarian preservation should be considered exclusively in patients who are fully informed about the risk of recurrence. For young women with LGESS who have undergone oophorectomy, careful follow-up and health management are imperative.

This study has some limitations. First, owing to the low incidence of LGESS, the analysis was confined to only six cases, which constrains the reliability of the results. Second, the retrospective nature of this study imposes inherent limitations. Third, interobserver agreement was evaluated for LGESS only. It, thus, remains unclear whether the observed reliability is specific to LGESS or represents a broader pattern across various uterine lesions. Finally, imaging findings were not compared with those of rare variant leiomyomas, which can present diagnostic challenges in differentiating them from LGESS. However, these issues have been extensively examined previously [3,8,13]. The strength of this study pertains to the referencing of these findings and corroborating their reproducibility in the presented cases.

## Conclusions

Differentiating a rare variant of leiomyoma from LGESS presents significant challenges based on the clinical and imaging findings. MRI features, including T2WI, DWI, and ADC values, provide valuable information for differentiation. In cases where LGESS is suspected, laparoscopic surgery with intraperitoneal morcellation should be avoided, and the indications for ovarian preservation must be rigorously evaluated.
